# Examining the Effect of Cu and Mn Dopants on the Structure of Zinc Blende ZnS Nanopowders

**DOI:** 10.3390/ma16175825

**Published:** 2023-08-25

**Authors:** Alexei Kuzmin, Inga Pudza, Milena Dile, Katrina Laganovska, Aleksejs Zolotarjovs

**Affiliations:** Institute of Solid State Physics, University of Latvia, Kengaraga Street 8, LV-1063 Riga, Latvia; inga.pudza@cfi.lu.lv (I.P.); milena.dile@cfi.lu.lv (M.D.); katrina.laganovska@cfi.lu.lv (K.L.); aleksejs.zolotarjovs@cfi.lu.lv (A.Z.)

**Keywords:** ZnS, Mn-doped ZnS, Cu-doped ZnS, X-ray absorption spectroscopy, reverse Monte Carlo, nanoparticles

## Abstract

It is known that doping zinc sulfide (ZnS) nanoparticles with Mn or Cu ions significantly affects their luminescent properties. Herein, we investigated how dopant atoms are incorporated into the structure of ZnS using X-ray diffraction and multi-edge X-ray absorption spectroscopy. The observed broadening of the X-ray diffraction patterns indicates an average crystallite size of about 6 nm. By analyzing the Zn, Mn, and Cu K-edge extended X-ray absorption fine structure (EXAFS) spectra using the reverse Monte Carlo method, we were able to determine the relaxations of the local environments around the dopants. Our findings suggested that upon the substitution of Zn by Mn or Cu ions, there is a shortening of the Cu–S bonds by 0.08 Å, whereas the Mn–S bonds exhibited lengthening by 0.07 Å. These experimental results were further confirmed by first-principles density functional theory calculations, which explained the increase in the Mn–S bond lengths due to the high-spin state of Mn${}^{2+}$ ions.

## 1. Introduction

Zinc sulfide (ZnS) is a wide-bandgap semiconductor that exists in two main crystallographic forms, cubic zinc blende (or sphalerite) and hexagonal wurtzite, with direct band gaps at room temperature of about 3.72 eV and 3.77 eV, respectively [1,2]. The wide range of ZnS applications is related to its ability to be manufactured in single crystal and powder forms, as well as in various nanostructures with different morphologies. These nanostructures include zero-dimensional configurations such as nanoparticles and quantum dots; one-dimensional structures such as nanowires, nanobelts, nanoribbons, nanosheets, nanotubes, nanocombs, and nanoawls; as well as two-dimensional thin films [1,2,3]. Additionally, the properties of ZnS can be varied by doping with transition metal or rare-earth ions [1,4,5,6].

The use of ZnS as a phosphor/scintillator [7,8,9,10,11] and in electroluminescence devices [12,13,14] has stimulated interest in its luminescent properties, which were reviewed in [15]. It was suggested that impurities and impurity-containing defects may play a key role in both nominally undoped and doped ZnS. Therefore, understanding how impurity atoms are incorporated into the ZnS structure is of both fundamental and applied interest. At the same time, obtaining such information using a diffraction technique is a non-trivial task due to a small concentration of dopant ions and, often, a nanosize of ZnS particles.

X-ray absorption spectroscopy (XAS) is a structural method that is element-selective and sensitive to low element concentrations when a fluorescence detection mode is used [16,17,18]. This makes it ideally suitable to study the local environment around impurities. Moreover, the simultaneous analysis of the extended X-ray absorption fine structure (EXAFS) spectra obtained at the absorption edges of elements composing the multicomponent material makes it possible to reconstruct their local environment within the framework of a single structural model using the reverse Monte Carlo (RMC) method [19,20]. As a result, accurate information on interatomic distances, bond strengths, and local structure distortions can be obtained [21].

In the past, the XAS method was applied to probe the local environment around dopants in ZnS in several works [22,23,24,25,26,27,28]. The main findings are summarized below.

The local structure around Mn luminescent centers was studied in Mn-doped ZnS nanocrystals with a size of 3–6 nm in [22]. It was found that the Mn ions substitute for the Zn ions and have an oxidation state close to 2+, whereas the mean bond length of Mn–S is slightly larger than that of Zn–S (2.34 Å) and decreases from 2.42 Å in the bulk sample down to 2.36–2.39 Å in the nanocrystals.

ZnS:Mn nanoparticles capped with a ZnO layer and annealed in a vacuum at temperatures up to 525 °C were studied in [23]. The annealing below 350 °C resulted in nanoparticles having the zinc blende phase and a size below 8.4 nm, whereas after annealing at 400 and 525 °C, a mixture of zinc blende and wurtzite phases was found with a larger crystallite size of about 16–17 nm. The Mn–S bond length of 2.394 Å was determined for the as-grown nanoparticles compared to 2.428 Å in the bulk.

Highly ordered Zn${}_{1-x}$Mn${}_{x}$S nanowires (*x* ranging from 0.01 to 0.3) with lateral dimensions of 3, 6, and 9 nm were synthesized within mesoporous SiO${}_{2}$ matrices and studied at the Mn K-edge in [24]. It was found that most Mn${}^{2+}$ ions randomly substitute Zn${}^{2+}$ ions inside nanowires while the amount of Mn at the surface is about 1–4%. The enlarged Mn–S bond lengths equal to 2.38–2.41 Å were observed in all nanowires.

Co-doped wurtzite-type Zn${}_{0.99-x}$Mn${}_{0.01}$Cu${}_{x}$S (*x* = 0, 0.003, 0.01) nanowires prepared by a hydrothermal method were studied at the Mn K-edge and Cu K-edge in [25]. The results indicate that both dopants are embedded in the ZnS lattice, replacing Zn${}^{2+}$ ions. Close results were obtained by the same group [26] for Mn-doped ZnS nanocrystals (10–15 nm) and nanorods (8–10 nm), synthesized by a solvothermal method using different solvents. Analysis of the EXAFS spectra gave the nearest interatomic distances of 2.42 Å for Zn–S bonds and 2.38–2.40 Å for Mn–S bonds.

Zn${}_{1-x}$Mn${}_{x}$S (*x* = 0.00, 0.01, 0.03, 0.05, 0.10) nanoparticles prepared by solvothermal synthesis were studied in [27]. The size of crystallites was estimated from the broadening of X-ray diffraction patterns and was among the smallest ones (1.5–1.9 nm). Only the Zn K-edge was studied by XAS and the estimated Zn–S bond lengths were about 2.30 Å compared to 2.33 Å in the bulk ZnS.

The local distortions around In${}^{3+}$ and Cu${}^{2+}$ dopants in synthetic sphalerite ZnS were recently probed in [28] using EXAFS spectroscopy combined with the RMC method, similar to the present study. Nevertheless, the RMC simulations in [28] were performed for isolated clusters without periodic boundary conditions that could potentially lead to less accurate numerical results. The values of interatomic distances within the Gaussian distribution model were found to be equal to 2.34 Å for the Zn–S bonds, 2.28–2.30 Å for the Cu–S bonds, and 2.45–2.46 Å for the In–S bonds.

Summarising the previous structural studies, it can be concluded that Zn–S and Cu–S bond lengths in the zinc blende phase vary by a few hundredths of an angstrom, with Cu–S bonds being slightly shorter. However, the scatter of bond lengths is larger for Mn–S bonds, which are also the longest. Therefore, further efforts are required to obtain more precise structural data, as well as to understand the origin of the differences between the different bonds.

**Figure 1:**
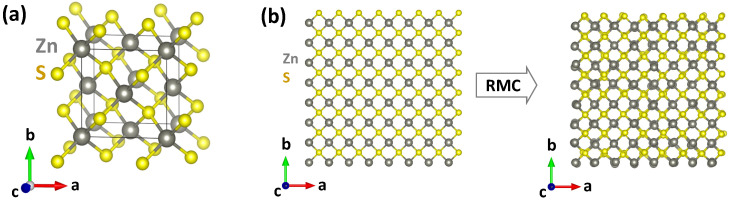
(**a**) Crystallographic structure of zinc blende ZnS. (**b**) An example of the initial and final structural models (supercells) for pure ZnS used in the RMC simulations. Crystallographic axes and atoms are indicated. See text for details.

In this study, we have used X-ray absorption spectroscopy to investigate how dopant atoms Cu and Mn are incorporated into the structure of ZnS nanoparticles with the zinc blende structure (Figure 1a). The RMC method was utilized to perform multi-edge EXAFS analysis, enabling us to determine structural models that were fitted simultaneously to two independently measured EXAFS spectra obtained from the same sample. The experimental results were complemented by first-principles simulations of structure relaxation around dopant atoms and provided us with additional information on the correlation between the ion size and the spin state of dopants.

## 2. Materials and Methods

### 2.1. Nanoparticle Synthesis and Characterization

Undoped and Mn-doped nanocrystalline zinc blende ZnS powders were synthesized using the microwave-assisted solvothermal (MWST) method as described in [29]. Briefly, ZnCl${}_{2}$ (purity 99.9%; Supelco, Bellefonte, PA, USA) and Mn(CH${}_{3}$COO)${}_{2}$·4H_2_O (purity 99.9%; Sigma Aldrich, St. Louis, MO, USA) salts taken in proper ratios were first dissolved in 45 mL ethylene glycol (purity 99.5%, Fisher Scientific, Waltham, MA, USA) to achieve a solution with 0.4 M total metal ion concentration. The product was mixed with a solution of 0.018 mol NaS${}_{2}$·9H_2_O (purity 98%; Acros Organics, Waltham, MA, USA) in 45 mL ethylene glycol. The MWST synthesis was performed in an inert atmosphere (N${}_{2}$, purity 99.999%; Linde AG, Pullach, Germany) at 200 °C for about 1.5 h using a Milestone synthWAVE T660 (Milestone Inc., Shelton, CT, USA) microwave reactor operated at a frequency of 2.45 GHz. After synthesis, the reaction product was cooled down to room temperature for 30 min, centrifugated, and washed with methanol. The obtained nanopowders were dried at 60 °C for 72 h.

Nanocrystalline zinc blende Cu-doped ZnS powder was synthesized using the hydtrothermal method. The first solution was prepared by dissolving ZnCl${}_{2}$ (0.197 M) in 45 mL of the deionized (DI) water and adding 0.2 mmol of CuSO${}_{4}$·5H_2_O (purity 97%; Sigma Aldrich, St. Louis, MO, USA) (0.003 M), along with sodium dodecyl sulfate (SDS, purity 98%; Fluka, Buchs, Switzerland) surfactant (0.01 M). The second solution was prepared by dissolving thiourea (CS(NH${}_{2}$)${}_{2}$, purity 99.9%; Carl Roth GmbH + Co. KG, Karlsruhe, Germany) (0.17 M) in 45 mL of DI water. Next, the first solution was poured into a Teflon-lined stainless-steel autoclave and the second solution was added dropwise. The hydrothermal reaction was carried out at 160 °C for 22 h with a heating rate of 5 °C/min in a laboratory oven. After the reaction, the powder was separated from the solvent by centrifugation and washed six times with 15 mL of DI water.

The Thermo Fisher Scientific Helios 5 UX (Waltham, MA, USA) scanning electron microscope (SEM) was used to study the elemental composition of all samples using energy-dispersive X-ray (EDX) spectroscopy. The lattice parameters and size of the synthesized nanopowders were determined by X-ray powder diffraction (XRD) using a Bragg-Brentano type θ–2θ benchtop diffractometer, Rigaku MiniFlex 600 (Rigaku Corp., Tokyo, Japan), equipped with a 600 W Cu anode (Cu Kα radiation) X-ray tube. X-ray diffraction patterns were measured at room temperature and analyzed using the Rietveld refinement method with the Profex 5.0.1 code [30].

### 2.2. X-ray Absorption Spectroscopy and Data Analysis

X-ray absorption spectra of pure ZnS and Mn/Cu-doped ZnS nanopowders were recorded at the DESY PETRA-III P65 Applied XAFS beamline [31]. The transmission detection mode with two ionization chambers was used for the Zn K-edge (9659 eV), whereas the total fluorescence detection mode with a passivated implanted planar silicon (PIPS) detector (CANBERRA Industries, Meriden, CT, USA) was employed for the Mn (6539 eV) and Cu (8979 eV) K-edges. The X-rays from an undulator source were monochromatized using a fixed-exit double-crystal Si(111) monochromator, and the harmonic reduction was achieved using an uncoated silicon plane mirror. The samples were prepared as pellets by mixing the nanopowders with cellulose and placed in a continuous flow liquid helium cryostat. The measurements were performed at a temperature of 10 K to reduce the contribution of thermal disorder.

The XAESA code [32] was utilized to extract the experimental extended X-ray absorption fine structure (EXAFS) spectra χ(k) (where *k* is the photoelectron wavenumber) above each absorption edge, following a conventional procedure [33]. The Fourier transforms (FTs) of the EXAFS spectra were calculated using a 10% Gaussian function to discern contributions from different coordination shells in *R*-space. It is important to note that the FTs of the EXAFS spectra were not corrected for the phase shift present in the EXAFS equation. As a result, the positions of peaks in the FTs are shifted to shorter distances compared to their crystallographic values.

The reverse Monte Carlo (RMC) method was used to extract structural information from the experimental EXAFS spectra. The RMC method is particularly suitable for studying metal-doped ZnS because it enables fitting a structural model simultaneously to two independently measured EXAFS spectra taken from the same sample, thereby enhancing the reliability of the model. The RMC simulations were performed using the EvAX code [34,35]. In this method, the difference between the Morlet wavelet transforms (WTs) [36] of the experimental and calculated configuration-averaged EXAFS spectra, available at one or more absorption edges, is minimized simultaneously in *k* and *R* space using the evolutionary algorithm (EA) [35]. The structural model (simulation box) represented a 4*a*× 4*a*× 4*a* supercell (Figure 1b) with periodic boundary conditions constructed based on the zinc blende lattice with the lattice parameter *a* = 5.406 Å, determined by XRD. The simulation box contained 256 Zn and 256 S atoms in pure ZnS, whereas 16 zinc atoms were substituted by Mn or Cu atoms in the doped ZnS. The dopant Mn/Cu atoms were distributed uniformly in the box to ensure that their local environment can relax independently during the RMC simulations. We simultaneously used 32 atomic configurations in the EA algorithm [35], and the atoms were allowed to displace from their equilibrium positions in the ideal zinc blende structure by less than 0.4 Å. Ten RMC/EA simulations were conducted for each experimental data set using different sequences of pseudo-random numbers. At each step of the RMC/EA simulation, the configuration-averaged EXAFS spectra were evaluated over all the absorbing atoms of the same type (Zn, Mn, or Cu) located in the simulation box. Ab initio real-space multiple-scattering FEFF8.5L code [37,38] was used to calculate the EXAFS spectrum for each atom employing the complex energy-dependent exchange–correlation Hedin–Lundqvist potential to account for inelastic effects [39]. The multiple-scattering effects up to the fourth order were included in the calculations [40,41,42].

### 2.3. First-Principles DFT Calculations

To investigate the impact of Mn or Cu doping on the ZnS lattice, first-principles density functional theory (DFT) calculations were performed using the linear combination of atomic orbitals (LCAO) method implemented in the CRYSTAL17 code [43]. All-electron triple-zeta valence (TZV) basis sets augmented by one set of polarization functions (pob-TZVP rev2) were employed for S, Mn, Cu, and Zn atoms [44]. The Monkhorst-Pack scheme [45] with an 8 × 8 × 8 k-point mesh was used to integrate the Brillouin zone. The self-consistent field (SCF) calculations were performed employing the hybrid M06 functional [46] with a tolerance of 10${}^{-10}$ for the total energy change.

The structural models were constructed based on the diffraction data for zinc blende ZnS [47] with the space group $F\bar{4}3m$ (No. 216). A primitive unit cell with two atoms (Zn and S) was used for pure ZnS, whereas an expanded primitive cell (supercell), 2 × 2 × 2 with 16 atoms (8 S, 7 Zn, and 1 Mn/Cu), was used for Mn/Cu-doped ZnS. The dopant atom was placed at the Zn 4*a*(0,0,0) site. The spin-polarized calculations were performed for fully relaxed geometry, providing information on the nearest interatomic distances and their change upon substitution of Zn by Mn/Cu.

## 3. Results and Discussion

The chemical composition of doped samples was determined using energy-dispersive X-ray (EDX) spectroscopy. It revealed approximately 0.8 at% Cu doping and 1.0 at% Mn doping (Table 1).

**Figure 2:**
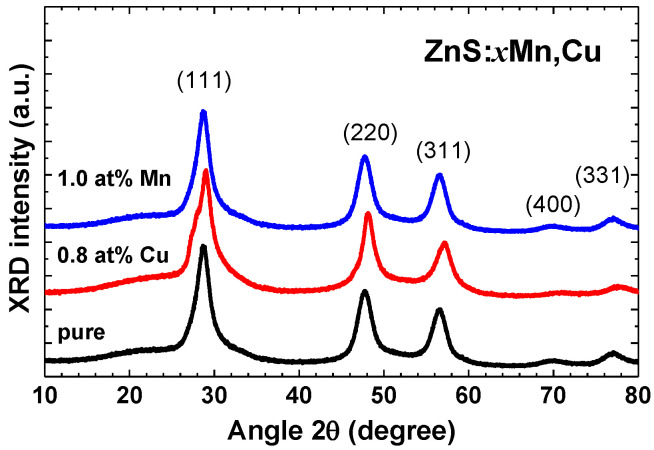
X-ray diffraction patterns of pure, 1.0 at% Mn-doped, and 0.8 at% Cu-doped zinc blende ZnS nanopowders. The main Bragg peaks are indexed.

X-ray diffraction patterns of pure and doped ZnS nanopowders are shown in Figure 2. The observed Bragg peaks were indexed using the cubic zinc blende phase [47,48] with the space group $F\bar{4}3m$ (No. 216). The broadening of the peaks indicates the nanocrystalline nature of all samples. The values of the lattice parameter *a* and crystallite size *d* were obtained through Rietveld analysis and are reported in Table 1. It is evident that all three samples exhibit similar lattice parameters and average crystallite size. The lattice parameter obtained by XRD was further used to construct the structural model for the RMC simulations of EXAFS spectra.

X-ray absorption spectroscopy provides structural information on the local environment around a particular element. This method is particularly useful when dopants are present in a compound. It allows for independent experiments to gather information about the main elements and dopants, which can then be combined during the RMC analysis. This combined approach enhances the reliability of the structural model extracted from the data [19,21].

**Figure 3:**
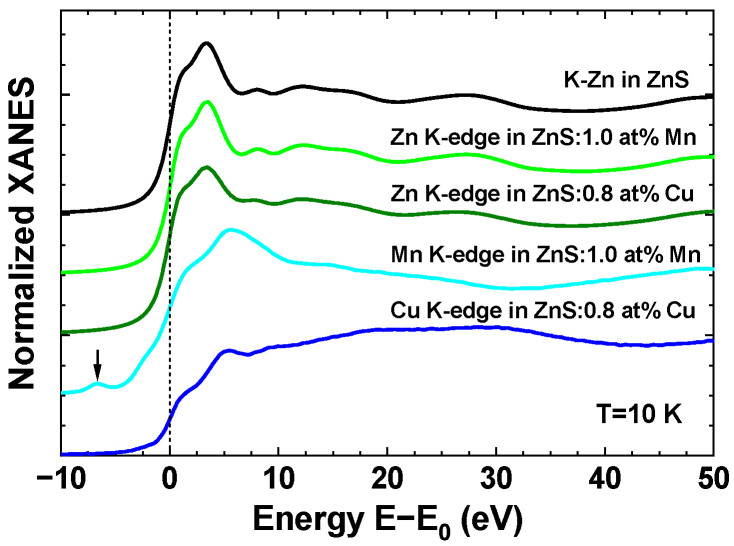
Experimental Zn, Mn, and Cu K-edge XANES spectra of pure, 1.0 at% Mn-doped, and 0.8 at% Cu-doped zinc blende ZnS nanopowders at *T* = 10 K. Curves are vertically shifted for clarity. The energy scale is set relative to the position $E_{0}$ of the maximum of the first derivative of the absorption coefficient. The pre-edge peak at the Mn K-edge is indicated with an arrow.

Normalized experimental X-ray absorption near edge (XANES) spectra of three samples measured at the Zn, Mn, and Cu K-edges at a temperature of 10 K are compared in Figure 3. Their energy scale was set relative to the position $E_{0}$ of the maximum of the first derivative of the X-ray absorption coefficient. Note that the shapes of the K-edge XANES spectra are close to those published in [49]. The main absorption edge is due mainly to the dipole-allowed transitions from the 1s core orbital of the metal atom to the continuum of p-like states above the Fermi level [50,51]. The weak pre-edge peak is also visible in the Mn K-edge XANES at about 7 eV below the main edge. Its origin is due to the transitions from the 1s core state to the 3d band hybridized with the 4p metal states [51,52]. The fine structure located above the absorption edge is determined mainly by the local atomic structure, and the main low frequency reflects a contribution from the nearest four sulphur atoms located in the first coordination shell of the absorbing metal atom. Note that the Zn K-edge XANES spectra are similar in three samples, suggesting the similarity of the local environment around zinc atoms. At the same time, the Mn and Cu K-edge XANES spectra deviate from the Zn K-edge spectra, indicating some change in the local structure. A close look at the XANES shapes allows one to observe a change in the frequency of the first shell contribution, which has a minimum at about 37 eV at the Zn K-edge, but at about 32 eV at the Mn K-edge and 44 eV at the Cu K-edge. This means that compared to the Zn–S interatomic distance in the first coordination shell, the Mn–S distance increases, but the Cu–S distance decreases. The numerical values of all distances obtained from the analysis of the EXAFS spectra are discussed below.

**Figure 4:**
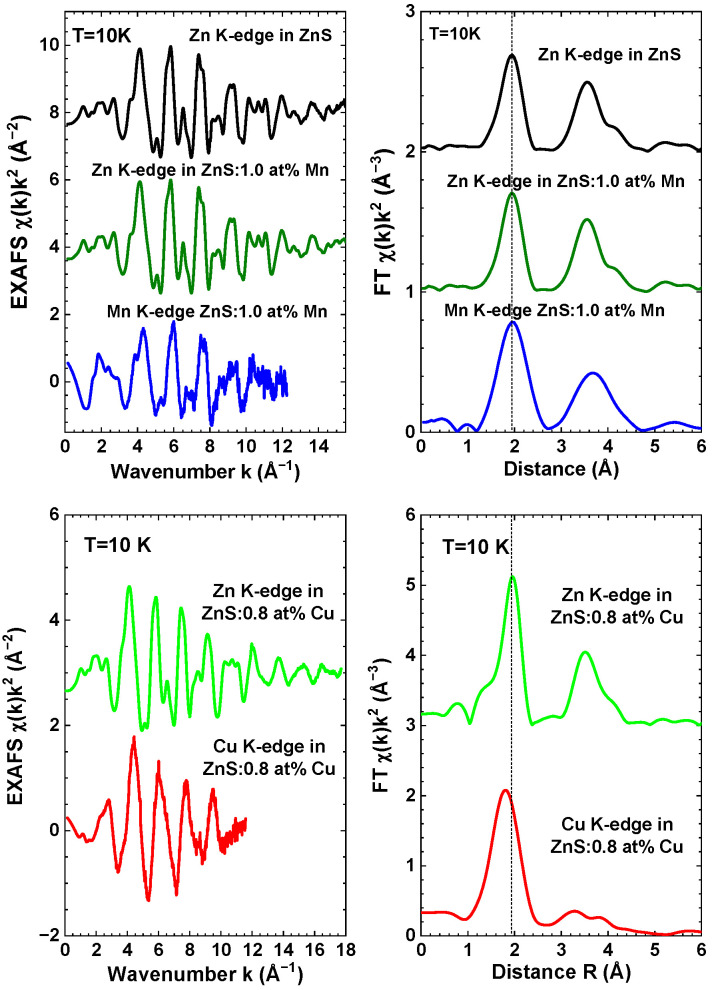
Experimental Mn, Cu, and Zn K-edge EXAFS spectra $\chi \left(k\right)k^{2}$ and their Fourier transforms (FTs) of pure, 1.0 at% Mn-doped, and 0.8 at% Cu-doped zinc blende ZnS nanopowders at *T* = 10 K. Curves are vertically shifted for clarity. Only moduli of FTs are shown. Vertical dashed lines are guides for the eye.

The experimental EXAFS spectra $\chi \left(k\right)k^{2}$ of pure and Cu/Mn-doped ZnS nanopowders, acquired at the Zn, Mn, and Cu K-edges, are depicted in Figure 4. It is worth noting that the range of the EXAFS spectrum at the Cu K-edge is limited due to the presence of the Zn K-edge, which is located just 680 eV higher in energy. The overall shape of the EXAFS oscillations, as well as their FTs, are similar for all three elements, indicating the similarity of their local environments. This confirms the substitution of zinc atoms by copper or manganese atoms. However, a slight difference in the position of the first peak at about 2 Å is specifically observed at the Cu K-edge. Additionally, noticeable variations in the shape of the second group of peaks within the range of 3–4 Å are visible. This suggests a relaxation of the local environment around the dopants.

**Figure 5:**
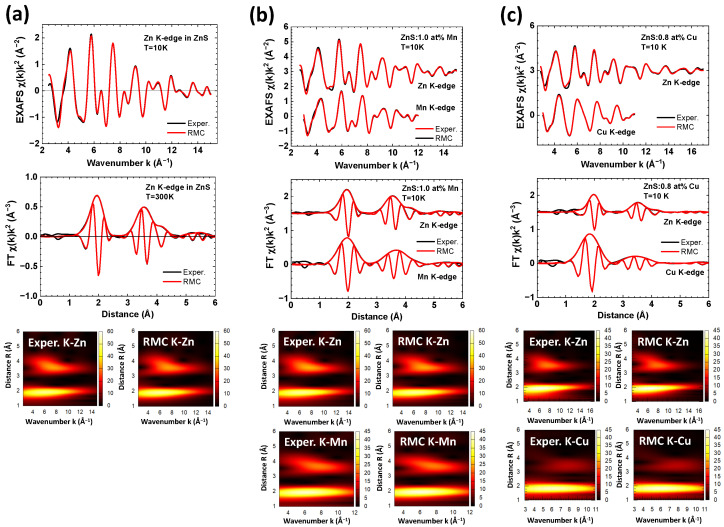
Experimental and RMC-calculated Zn, Mn, and Cu K-edge EXAFS spectra $\chi \left(k\right)k^{2}$ and their Fourier and Morlet wavelet transforms for pure (**a**), 1.0 at% Mn-doped (**b**), and 0.8 at% Cu-doped (**c**) zinc blende ZnS nanopowders at *T* = 10 K.

**Figure 6:**
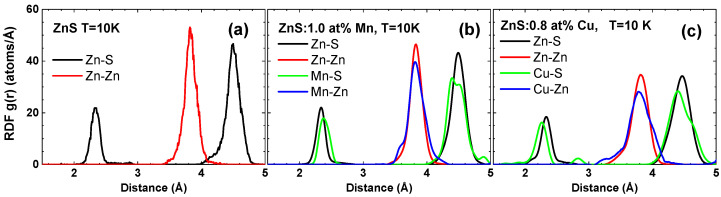
Radial distribution functions (RDFs) g(r) for Zn–S, Zn–Zn, Mn–S, Mn–Zn, Cu–S, and Cu–Zn atom pairs in pure (**a**), 1.0 at% Mn-doped (**b**), and 0.8 at% Cu-doped (**c**) zinc blende ZnS nanopowders at *T* = 10 K.

More detailed information on the structure of pure and doped ZnS was extracted through the RMC analysis of the EXAFS spectra, as reported in Figure 5. A good agreement was achieved between the experimental and calculated EXAFS spectra in *k*, *R*, and (*k*, *R*) space. The partial radial distribution functions (RDFs), calculated from the coordinates of atoms in the simulation box, are shown in Figure 6. These RDFs correspond to the zinc blende structure, where each metal atom is surrounded by 4 sulphur atoms in the first coordination shell (with the peak at ∼2.4 Å), 12 metal atoms in the second shell (with the peak at ∼3.8 Å), and 12 sulphur atoms in the third shell (with the peak at ∼4.5 Å).

The presence of both dopants affects the local structure around zinc atoms, resulting in the broadening of the Zn–S and Zn–Zn RDFs. The effect of copper doping is more pronounced compared to manganese doping. Furthermore, the local environment around copper and manganese atoms exhibits distinct relaxation patterns. It is evident that the relative position of the first peak is shifted towards shorter distances for the Cu–S RDF, whereas it shifts towards longer distances for the Mn–S RDF. To understand this behaviour, one can refer to the empirically derived effective ionic radii of the three metal ions [53]: *r*(Zn${}^{2+}$) = 0.60 Å, *r*(Cu${}^{2+}$) = 0.57 Å, *r*(Mn${}^{2+}$[HS]) = 0.66 Å (where HS denotes the high-spin state). These values correlate well with the average metal–sulphur interatomic distances calculated from the RDFs: *R*(Zn–S) = 2.33(2) Å, *R*(Cu–S) = 2.25(2) Å, and *R*(Mn–S) = 2.40(2) Å.

To support these findings, we conducted first-principles DFT-LCAO calculations using the CRYSTAL17 code [43] on pure and doped zinc blende ZnS. The supercell approach was employed to introduce dopant atoms into the zinc blende structure, and both the low-spin (LS) and high-spin states of manganese ions were simulated. The calculated interatomic distances in the fully relaxed geometry are equal to *R*(Zn–S) = 2.34 Å, *R*(Cu–S) = 2.31 Å, *R*(Mn[LS]–S) = 2.29 Å, and *R*(Mn[HS]–S) = 2.38 Å. These values align with the EXAFS results and reproduce the ion size dependency of interatomic metal–sulphur bond lengths in ZnS. Additionally, the calculations indicate that the size of the manganese ion in the high-spin state is about 0.1 Å larger than in the low-spin state.

The mean-squared relative displacements (MSRDs) $\sigma^{2}$ at *T* = 10 K obtained using the RMC simulations are reported in Table 2. It is important to note that the MSRD values for Zn–S1, Zn–Zn2, and Zn–S3 atom pairs corresponding to the nearest three coordination shells are close, within errors, in pure ZnS and in the ZnS sample doped with 1.0 at% Mn, but larger in the ZnS sample doped with 0.8 at% Cu. The disorder around the larger Mn${}^{2+}$ ions is only slightly larger than that around the smaller Zn${}^{2+}$ ions due to some relaxation of the structure. Meanwhile, the local environment around the smaller Cu${}^{2+}$ ions undergoes stronger relaxation, leading to even larger values of MSRDs for the Cu–S1, Cu–Zn2, and Cu–S3 atom pairs.

## 4. Conclusions

The effect of Cu and Mn doping on the structure of zinc blende ZnS nanopowders with a crystallite size of about 6 nm was studied using X-ray diffraction and X-ray absorption spectroscopy (EXAFS/XANES). The experimental results were complemented by first-principles DFT-LCAO calculations.

We demonstrate that a multi-edge analysis of EXAFS spectra, employing the reverse Monte Carlo method, provides a reliable structural solution that is sensitive to the relaxations of the local environment surrounding dopant ions. Our results confirm that both Mn and Cu dopants substitute Zn ions. An increase in disorder within the ZnS structure was observed upon doping, especially around the dopant ions, and this effect is more pronounced when doping with copper. The relaxation of sulphur atoms in the first coordination shell of metals depends on the size of the metal ion. Consequently, the interatomic distances in the first coordination shell, as determined by EXAFS, follow the sequence: *R*(Mn–S) >*R*(Zn–S) >*R*(Cu–S). First-principles calculations suggest that the largest *R*(Mn–S) distance and, thus, manganese ion size, can be attributed to the high-spin state of Mn${}^{2+}$ ions. Thus, combining EXAFS results with first-principles calculations enables the discrimination of the spin state of the dopant ion.

## Figures and Tables

**Table 1:** The lattice parameters and crystallite size for pure, 0.8 at% Cu-doped, and 1.0 at% Mn-doped zinc blende ZnS nanopowders.

*x* (at%)	Lattice Parameter *a* (Å)	Crystallite Size *d* (nm)
0	5.406 (2)	5.8 (2)
0.8 (Cu)	5.400 (2)	6.0 (2)
1.0 (Mn)	5.406 (2)	5.8 (2)

**Table 2:** The mean-square relative displacements (MSRDs) $\sigma^{2}$ for the first three coordination shells of metal ions at *T* = 10 K obtained using the RMC simulations for pure, 1.0 at% Mn-doped, and 0.8 at% Cu-doped zinc blende ZnS nanopowders.

	MSRD $\sigma^{2}$ (Å${}^{2}$)	
**Atom Pair**	**ZnS & ZnS:Mn**	**ZnS:Cu**
Zn–S1	0.005 ± 0.003	0.009 ± 0.003
Zn–Zn2	0.010 ± 0.003	0.020 ± 0.003
Zn–S3	0.016 ± 0.003	0.020 ± 0.003
Mn–S1	0.006 ± 0.003	
Mn–Zn2	0.018 ± 0.003	
Mn–S3	0.023 ± 0.003	
Cu–S1		0.010 ± 0.003
Cu–Zn2		0.039 ± 0.003
Cu–S3		0.028 ± 0.003

## Data Availability

The data presented in this study are available on request from the corresponding author.

## Abbreviations

The following abbreviations are used in this manuscript:

DFT	density functional theory
DI	deionized
EA	evolutionary algorithm
EDX	energy-dispersive X-ray
EXAFS	extended X-ray absorption fine structure
FT	Fourier transform
HS	high-spin
LCAO	linear combination of atomic orbitals
LS	low spin
MSRD	mean-squared relative displacements
MWST	microwave-assisted solvothermal
NP	nanoparticle
RDF	radial distribution function
RMC	reverse Monte Carlo
RT	room temperature
SCF	self-consistent field
SDS	sodium dodecyl sulfate
SEM	scanning electron microscope
TZV	triple-zeta valence
WT	wavelet transform
XANES	X-ray absorption near edge
XAS	X-ray absorption spectroscopy
XRD	X-ray diffraction
